# The Physician

**Published:** 1876-04

**Authors:** 


### HALL'S JOURNAL OF HEALTH.

**Our Legitimate Scope is almost boundless: for whatever begets pleasurable and harmless feelings, promotes Health; and whatever induces disagreeable sensations, engenders Disease.**

WE AIM TO SHOW HOW DISEASE MAY BE AVOIDED, AND THAT IT IS BEST, WHEN SICKNESS COMES, TO TAKE NO MEDICINE WITHOUT CONSULTING A PHYSICIAN.

APRIL, 1876.

### THE PHYSICIAN.

NEXT to the clergy, the educated physician is the greatest benefactor and conservator of the race, performs more personal service without fee or reward, and at his own individual expense, than any other class in society. Very much that doctors do is not appreciated by the great public; is never dreamed of by the masses. The common feeling toward a medical man is that he is expected to come to us when we send for him, to know what is the matter with us, what we need, and, after we get well, to send his bill, receive payment, and the whole is settled. But in all this, his solicitudes; the balancing of his hopes and fears; the comparisons he has to make in his own mind; the judgments he has to form; and the responsibilities he has to assume, singly and alone, without counsel, without sympathy—these are not taken into account; money could not pay for them.

There is another item which does not enter into the imagination of one man in a thousand. New forms of accidents are constantly occurring; new diseases are every now and then manifesting themselves for the first time in human history—sometimes so obscure, so singular, so unaccountable, that the public are stricken with alarm, often approaching absolute terror. Just before the cholera reached our shores in 1832 and '33, the feeling of the multitude was to run away from it; when it did come, many died from the effect of abject, unresisting fear. The physician went to meet it, to investigate its nature and report upon it, if he ever returned, that his brethren might know how to handle it and save the people. But did the public applaud? did they vote to him the freedom of our great cities?

did they honor him with public ovations, and receive him with flowers and songs and the rejoicings of the populace? Did any man or body of men offer to pay him for his time, his trouble, his risk, or even to refund the actual outlays of his journey. Not a mill toward that object was contributed by a single human being.

It may interest the inquiring reader to know the mode of procedure in reference to new diseases; whether attacking individuals, families, neighborhoods, or a whole nation. The main points to be ascertained are the causes, the symptoms, the nature, and the cure of the malady. If any one of these can be determined with reasonable certainty, all the others are pretty sure to follow sooner or later. In reference to the Asiatic cholera, one of the first impressions sent to us from across the Atlantic was, that persons in apparent perfect health were suddenly attacked and died in a few hours, in spite of all and every thing that could be done. The experienced physician had to look at it just as it presented itself; he seized hold of the great predominant symptom; that which overshadowed all others, and which was always present, never absent in one single case in all the millions previously reported; that was the "looseness" or thin, light-colored watery passages. This being the case, it began to be inquired how long that symptom had been observed; when was the looseness first noticed. In other words, health consists in one action of the bowels in each twenty-four hours—less, is disease; more, is disease. The patient was then desired to note the time when he first began to have more than one daily action of the bowels. It was then ascertained, by comparing notes, that in no single case of any intelligent and observing person, did it happen that the looseness came on suddenly; but its commencement was traced back from one to two, three, four, or more days; and the conviction was settled upon, as a general rule, that an actual attack of cholera never presen.ed itself until the bowels had been acting more than once in twenty-four hours for several days; later on it was determined that the rule proved be considered universal. In some cases the unobservant, the uneducated, or the imbecile, reported differently, but the testimony of the cultivated and the reflecting, backed by the personal experience of those physicians who were themselves attacked with the disease, had to be received as conclusive. It was not long before this fact was heralded through every medical publication in the land; was reduced to popular form, and handed to the newspapers, embodying the great fact, that cholera was, in all cases, ushered in by "premonitory symptoms," which, if promptly attended to on general principles, were as easily, as safely, and as certainly removed, as the very commonest disease known; in fact, it was soon found out, that if a man seeing himself have two or three actions of the bowels in twenty-four hours, in cholera times, would only go to bed and keep quiet in body and mind in a cool, clean, well-aired room, the disease would be averted and the man be well in a day or two without a single atom of any kind of medicine whatever. This great fact has remained incontrovertible to the present hour.

But it will be of greater interest to detail a case of very recent occurrence in the army; and there is something of the fearful about it. Three regiments of soldiers—the Forty-third, Forty-fifth, and Fifty-first Massachusetts Volunteers—were encamped together as near as possible. Within a month, an unheard-of disease appeared among them. The soldier was suddenly attacked without warning, with a chill, and died within twelve hours, almost as certainly as attacked. The Forty-fifth had five cases, all fatal; the Fifty-first had seventeen cases, not one recovered; the Forty-fourth had twenty cases, of which twelve died; the remaining eight lingered, but did not recover. The instinct of any community under such circumstances would have been to run wildly away; this was not possible for soldiers. The surgeon had to look at the danger face to face, and do all that was possible, first to find out the cause and then remove it. A common mind might have looked into matters for a month or a year, by which time every solitary man would have made his last bivouac under the clods of the field. Not so with the surgeon; his first care was to notice if one regiment suffered more in proportion to its numbers than another; and if so, what circumstances were there in the regiment which suffered most, different from those in the regiment the least afflicted. The Forty-third regiment did not report a single case, yet these three regiments were close together. All the Forty-third lived in tents; the other two lived in wooden barracks. But soldiers had lived in wooden barracks thousands of times before, without any such disease appearing; still there was the great difference staring the surgeon in the face. The linen tents gave life; the wooden barracks spread a deadly disease. These barracks were made of hard, green pine timber. It was obvious, then, that the first great step to be taken was to order a change of quarters, and thus see if barracks made of hard, green pine lumber was adequate to the generation of such a deadly malady. As soon as the quarters were changed, the disease forthwith disappeared. The Fifty-first regiment were allowed to remain longer in their barracks; the disease meanwhile spreading itself, when they, too, shifted their quarters, and the malady suddenly disappeared. There can be no two opinions in a case like this; the cause of the sickness was living at that season of the year in houses made of hard, green pine-wood; and the cause having been demonstrated, it only remained to remove it. This is the highest, the noblest aim of the physician, to prevent disease; and who shall not say, that is a diviner skill than to cure?

But a burning shame would attach to the educated practitioner who would be satisfied with this attainment; hence in pity for the ignorant who might hereafter inadvertently expose themselves to the causes of disease, or be compelled to such exposure by those who had authority over them, it is important to find out the nature of the ailment, which often gives a clue to the remedy; the quickest way of doing this is to examine the dead body and see what parts of it are affected in a manner different from one which had perished in perfect health, by accident. In this case, several parts of the body were in a healthy condition; other parts were affected as they might have been in several common diseases; but some of these parts were affected in some cases but not in all. It was necessary then, as a point of departure, to notice if any part of the body of one dying of this strange disease, was always affected, and affected in the same way. This would be specific; it would be a foundation-stone, the "head of the corner." It was soon found with great uniformity that the membrane which invests, or lines the brain was inflamed, and as the spinal cord is but a prolongation of the brain, this inflammation was traced all along its course to its extremest distance, at the very tip end of the seat. Now, when it is remembered that if a single organ or lobule of the brain is inflamed, even a single square inch of it, the man is crazy as to that particular organ; when it is remembered further, that if a single inch of the spinal marrow is inflamed, it disorders every part of the human body which is supplied with nerves from that particular section, it can be readily seen how naturally it would follow that the whole system would so soon perish, when the entire brain and the entire spinal column, was in an actively diseased state in this new malady.

This disease is so recent, and of such a nature, that time has not been allowed to ascertain more than three prominent points: first, the cause; second, the seat; third, no treatment is of any avail. Further advances, however, will doubtless be made in due time, and published to the world.

This article has been written to give the general reader some idea, some better appreciation of what the practice of medicine really is, and how it is that a man learns to be a doctor; that "doctoring," as it is sometimes termed, is not to be taken up like a trade; it is not a thing to be learned in all its parts like the making of a dress, or the construction of an engine. A man may learn to make a box in an hour, and he may continue to make other boxes like it to the end of time; and all the *progress* he makes is in mechanical handiness, so that he shall make boxes hereafter in a neater manner and in a shorter time. The physician does not become master of his art by learning to cure one disease, by giving a particular drug; and then learn to cure another disease, by giving another medicine, until he has gone through all maladies yet known to man. He can't learn to be a successful physician by reading books; it is an impossibility. The very first case a young gentleman is called to, the day after he receives his diploma, is just as likely as not to have an element or circumstance connected with it unlike any he ever read or saw or heard of. But we are not then to conclude that he is powerless; that he is like a ship at sea without rudder or sail; that he knows no more what to do than any body else; very far from it. He has been indoctrinated into the general principles of medicine, and he knows he has to observe, compare and decide; observe the symptoms, compare them with others which he has seen, read or heard of, and then by the exercise of a calm judgment, determine what remedies are applicable to that particular character and class of symptoms. Thus an educated physician may intelligently prescribe for an ailment new to him, with as reasonable hopes of success as a man skilled in the mechanic arts can fabricate a piece of furniture or a machine, the like of which never entered the brain of mortal man before, according to the description given him by the person desiring it to be made. He does this by the practical application of certain known principles of adaptation, of cause and effect; and only such a man is entitled to be considered as master of his trade. And he only becomes a skillful and successful physician who practices his profession, not by the administration of medicines for symptoms, according to the book, but by the apt application of certain well-established principles to the particular case in hand.

It may be useful in this connection to impress one wholesome truth on the popular mind, which, by the way, sadly needs enlightenment in this particular direction. It is a part of the education of the medical profession, and which no honorable man ever ignores, to have all things in common; every thing that is new as to the cause, character and cure of disease. As soon as an educated physician finds a new remedy for an ailment, he communicates it to the medical journals, and within a year it is known all over the civilized world; the result is, his brethren in all nations have the benefit of his discovery; they do the same thing in return, and thus the physician who keeps up with the times, has not only the advantage of his own experience, but that of all the scientific and practical members of the profession throughout the world. But if by chance the charlatan or the quack becomes acquainted with a valuable remedy for a particular disease, instead of generously throwing it open to the whole world for the benefit of suffering humanity, he locks it up in his own bosom, hoards the precious treasure as the miser does his gold, and doles it out in the shape of a "patent medicine" to those only who can pay for it; others may die for aught he cares; and those who countenance and aid and abet him, by giving him certificates for publication, do but make themselves partakers of his meanness, and justly merit the contempt of the humane and the good of every land.

A RELIGIOUS newspaper in Philadelphia announces in its column of *General News*, "Not more necessary for the preservation of life on the battle field is a complete armor, than is a bottle of * * * pain killer to those who are suffering from acute bodily pain."

The above is neither new nor true, and that it should be placed among items of news, when it is paid for as an advertisement, is to our mind not good morals in any paper, secular or religious.

